# Impact of preoperative biliary drainage on postoperative complications and prognosis after pancreaticoduodenectomy: A single-center retrospective cohort study

**DOI:** 10.3389/fonc.2022.1037671

**Published:** 2022-11-10

**Authors:** Dong Wang, Huajun Lin, Chengjian Guan, Xiaodong Zhang, Peixin Li, Chenglin Xin, Xiaobao Yang, Zhewen Feng, Yiyang Min, Xiaozhe Gu, Wei Guo

**Affiliations:** ^1^ Department of General Surgery, Beijing Friendship Hospital, Capital Medical University and National Clinical Research Center for Digestive Diseases, Beijing, China; ^2^ Department of Comprehensive Surgery, Beijing Friendship Hospital, Capital Medical University and National Clinical Research Center for Digestive Diseases, Beijing, China

**Keywords:** preoperative biliary drainage, obstructive jaundice, pancreaticoduodenectomy, surgery-related complications, survival prognosis

## Abstract

**Background and objectives:**

Obstructive jaundice is common in patients with pancreaticobiliary malignancies. Preoperative biliary drainage (PBD) can alleviate cholestasis; however, no consensus has been reached on the impact of PBD on the incidence of surgery-related complications and patient survival. This study aimed to evaluate the effect among patients treated with PBD.

**Methods:**

This retrospective study examined the clinical and follow-up prognostic data of 160 patients with pancreaticobiliary malignancies who underwent pancreaticoduodenectomy (PD) at Beijing Friendship Hospital, Capital Medical University, from January 2016 to July 2020. Outcomes were compared between patients who underwent PBD (PBD group) and those who did not (control group). Changes in biochemical indicators were evaluated before and after drainage in the PBD group. Between-group differences in inflammatory indicators after PD were assessed using the Wilcoxon signed-rank test. Postoperative complications were classified according to the Clavien-Dindo classification system. The effects of PBD and biliary drainage efficiency on postoperative complications were evaluated using the chi-square test and binary logistics regression. The Kaplan-Meier analysis was used for between-group comparison of survival analysis. Univariate and multivariate regression analyses were performed to identify prognostic factors of survival.

**Results:**

Total 160 patients were enrolled,the mean age of the study sample was 62.75 ± 6.75 years. The distribution of pancreaticobiliary malignancies was as follows: 34 cases of pancreatic head cancer, 61 cases of distal bile duct cancer, 20 cases of duodenal papilla cancer, 39 cases of duodenal ampullary cancer, and 6 cases of malignant intraductal papillary mucinous neoplasm (IPMN). PBD was performed in 90 of the 160 patients, with PBD performed using an endoscopic retrograde cholangiopancreatography (ERCP) approach in 55 patients and with percutaneous transhepatic cholangiography (PTC) used in the remaining 35 cases. The mean duration of drainage in the PBD group was 12.8 ± 8.8 days. The overall rate of complications was 48.05% (37/77) in the control group and 65.55% (59/90) in the PBD group with non-significant difference (χ2 = 3.527, p=0.473). In logsitics regression analysis, PBD was also not a risk factor for postoperative complications OR=1.77, p=0.709). The overall rate of postoperative complications was significantly higher among patients who underwent PBD for >2 weeks (χ2 = 6.102, p=0.013), with the rate of severe complications also being higher for this subgroup of PBD patients (χ2 = 4.673, p=0.03). The overall survival time was 47.9 ± 2.45 months, with survival being slightly lower in the PBD group (43.61 ± 3.26 months) than in the control group (52.24 ± 3.54 months), although this difference was not significant (hazard ratio (HR)=0.65, p=0.104).

**Conclusion:**

In patients with malignant biliary obstruction, PBD does not affect the incidence of postoperative complications after pancreaticoduodenectomy nor does it affect patient survival. Prolonged biliary drainage (>2 weeks) may increase the incidence of overall postoperative complications and severe complications.

## Introduction

Ampullary malignancies, such as pancreatic head, duodenal papilla, and distal bile duct cancers, are common malignancies of the pancreaticobiliary system (1, 2). The clinical efficacy of treatment for these cancers, however, is low, with the prognosis generally being poor (1, 2). Moreover, malignant biliary obstruction, leading to jaundice due to poor bile drainage caused by compression of the bile duct, is common in these patients (3). Worsening of cholestasis is associated with abnormal liver function, electrolyte disturbance, and decreased nutritional status, which may have a negative impact on patients’ tolerance for surgery and increase the incidence of postoperative complications (4, 5). Accordingly, preoperative biliary drainage (PBD) has become an important clinical component of the presurgical management of patients with ampullary malignancies (6). However, although extensive studies have been conducted in this regard at various clinical centers worldwide, there is currently no consensus as to whether PBD affects the incidence of post-pancreaticoduodenectomy complications and patient survival.

To address this knowledge gap, this retrospective cohort study aimed to evaluate the impact of PBD on the incidence of surgery-related complications and patient survival after pancreaticoduodenectomy (PD).

## Material and methods

### Statement of ethics

The methods of our study were approved by the ethics committee of Beijing Friendship Hospital, Capital Medical University (approval number: 2022-P2-104-01). This study complies with the Declaration of Helsinki, and all patients obtained informed consent before being included in this clinical study.

### Study sample

In this retrospective cohort study, the study sample included 160 patients with ampullary malignancies who underwent PD from January 2016 to July 2020 at the Beijing Friendship Hospital of Capital Medical University.

The included patients consisted of 94 males and 66 females, with an average age of 62.75 ± 6.75 years. They were divided into a PBD group (n=90) and a control group (n=70) according to whether they underwent PBD. The inclusion criteria were as follows: (1) ampullary malignancies (including pancreatic head, distal bile duct, duodenal papilla, and duodenal ampulla malignancies) as well as malignant intraductal papillary mucinous neoplasm (IPMN); (2) absence of distant organ metastases confirmed on preoperative assessment; and (3) clear pathological diagnosis and complete clinical data. The exclusion criteria were as follows: (1) PD combined with resection of other organs; (2) preoperative neoadjuvant therapy; (3) history of biliary drainage prior to admission; and (4) patient refusal to participate and/or incomplete clinical data. Based on the experience of our center,the indications for preoperative biliary drainage in patients with periampullary cancer include: cholangitis secondary to biliary obstruction, patients with severe pruritus,comorbidities requiring present the work - up and coagulopathy. In consideration of the different clinical features of patients with different pathological types, the patients were divided into pancreatobiliary type and intestinal adenocarcinoma type according to histology.

### Data collection

The following clinical data were retrospectively extracted from patients’ medical records for analysis: sex, age, body mass index (BMI), American Society of Anesthesiologists (ASA) classification, preoperative tumor markers, biochemical indicators, surgical approach, intraoperative blood loss volume, pancreaticojejunal anastomosis, and pathologic tumor type. PD-related complications considered in this study were pancreatic fistula, biliary leak, intra-abdominal bleeding, gastrointestinal bleeding, intra-abdominal infection, gastric emptying disorder, pulmonary infection, cardiopulmonary insufficiency, and pleural effusion, with the severity of these complications being determined according to the Clavien-Dindo classification system (7). Complications with a Clavien-Dindo grade ≥3 were considered severe. Based on the consensus of the International Study Group on Pancreatic Surgery, pancreatic fistula was defined as a drainage fluid amylase level >3-fold the upper limit of normal blood amylase level at ≥ 3 days postoperatively, along with related clinical symptoms, such as abdominal pain. The neutrophil-lymphocyte ratio (NLR) and C-reactive protein to albumin ratio (CAR) on postoperative days 1 and 3 were used as indicators of the systemic inflammatory status. In our center, patients were followed up every 3 months in the outpatient or internet clinic during the first postoperative year and every 3-6 months in the outpatient or internet clinic during the 2nd to 3rd year after surgery. Every 6 months in the outpatient,internet clinic or telephone follow-up continued for 3 years after surgery until death. In this study, 150 patients were followed up regularly, 10 patients were lost to follow-up, and the mean follow-up time was 47.9 months.

### PBD and PD methods

Methods for preoperative jaundice reduction included endoscopic biliary stenting (ENBD), endoscopic retrograde cholangiopancreatography (ERCP), percutaneous transhepatic cholangiography drainage (PTCD), or bile duct stenting, as appropriate for each patient. All procedures were performed by experienced endoscopists in the Gastroenterology Department at our hospital. In order to clarify the effect of different biliary drainage methods on postoperative complications, patients in the PBD group were divided into antegrade group and retrograde group according to the biliary drainage method in subgroup analysis. Biliary drainage *via* PTC was defined as the anterograde group, and biliary drainage *via* ERCP was defined as the retrograde group.In all patients, PD was performed *via* an open or laparoscopic approach by chief surgeons who had extensive surgical experience. Regional lymph node dissection was routinely performed. Digestive tract reconstruction was performed using the classical Child’s method, with either a mucosa-to-mucosa pancreatojejunostomy anastomosis or other anastomosis approach, as appropriate for each patient.

### Statistical analysis

Baseline patient information is presented as categorical or continuous variables, as appropriate, with between-group differences evaluated using the chi-squared test or independent sample t-test for categorical or continuous variables, respectively. Both PBD-induced changes in biochemical indicator levels and the impact of biliary drainage duration on the efficiency jaundice reduction were assessed using Wilcoxon signed-rank test and graphically presented (ggplot2, R package, version 3.3.3). The distribution of the efficiency of jaundice reduction for different PBD durations was graphically presented using ridge plots (ggridges, R package, version 0.5.3 R Foundation for Statistical Computing,Vienna, Austria. URL https://www.R-project.org/) with the efficiency of jaundice reduction on the rate of postoperative complications evaluated using the chi-squared test. The rate of postoperative complications was compared between the PBD and control groups, including a between-group comparison in postoperative inflammatory indicators using a Mann-Whitney U test and graphical representations (ggplot2, R package, version 3.3.3). For the occurrence of postoperative complications, we also conducted binary logistics regression analysis to eliminate confounding factors. Survival analysis was performed using the R package survival program (version 3.2-10) and graphically presented (survminer, R package, version 0.4.9). All analyses were performed using SPSS (version 26.0) and R (version 3.6.3), with a P-value <0.05 considered statistically significant.

## Results

### General information

Of the 160 patients enrolled in the study, 70 were included in the control group and 90 in the PBD group. In the PBD group, 55 patients were treated with endoscopic nasobiliary drainage (EBND) or biliary stents placed through ERCP and 35 with PTCD. No significant differences were noted between the PBD and control groups in baseline clinical characteristics, including sex, age, BMI, ASA classification, preoperative CA199 level, preoperative aspartate aminotransferase (AST), preoperative alanine aminotransferase (ALT), preoperative prothrombin time (PT), history of upper abdominal surgery, surgical approach, surgical duration, intraoperative blood loss, pancreaticojejunal anastomosis, pathologic type, and tumor grade (all p > 0.05). In the last laboratory examination before surgery, total bilirubin (TB) and direct bilirubin (DB) levels were higher in the PBD group than in the control group, while albumin level was significantly lower in the PBD group than in the control group (all p < 0.05, **Table 1**).

**Table 1:** Demographic and clinical characteristics of the patients at baseline.

	Control group (n = 70)	PBD group (n = 90)	statistic (χ2 or t value)	p value
Gender				
Male	39	55	0.473	0.491
Female	31	35		
Age, years				
<65	47	56	0.415	0.519
≥65	23	34		
Body-mass index, kg/m²				
≤24	35	50	0.488	0.484
>24	35	40		
ASA classification				
I-II	41	48	0.437	0.508
III-IV	29	42		
CA199, U/ml				
≤35	27	27	1.293	0.255
>35	43	63		
Laboratory Examination(Mean±SEM)				
TB, µmol/L	76.71±10.76	131.95±11.24	-3.475	0.001
DB, µmol/L	41.39±6.75	76.04±6.91	-3.522	0.001
AST, U/L	80.75±9.38	80.42±7.35	0.028	0.977
ALT, U/L	123.37±17.87	106.50±8.14	0.859	0.392
ALB, U/L	37.48±0.58	35.48±0.43	2.835	0.005
PT, s	12.34±0.66	12.09±0.12	0.405	0.686
History of upper abdominal surgery				
No	67	84	0.42	0.516
Yes	3	6		
History of chronic disease				
1	57	77	0.492	0.482
≥2	13	13		
Surgical approach				
LPD	53	65	2.4842	0.288
OPD	15	17		
LPD convert to OPD	2	8		
Operation time(min)	245.13±11.08	258.77±9.55	-0.934	0.352
Blood loss(ml)	382.85±43.68	462.77±43.26	-1.281	0.202
Total hospital stay(days)	28.21±1.42	38.35±2.15	-3.92	<0.001
Pancreaticojejunal anastomosis				
mucosa-to-mucosa	46	65	0.784	0.375
none mucosa-to-mucosa	24	25		
Pathologic type				
pancreatic head cancer	16	18	8	0.091
distal bile duct cancer	23	38		
duodenal papilla cancer	12	8		
duodenal ampulla cancer	14	25		
invasive IPMN	5	1		
Differentiation				
Well/Moderate	45	51	1.193	0.274
Poor	25	19		

The p values of the statistical results of these factors were all less than 0.05, with significant statistical difference.

### Efficiency of preoperative jaundice reduction and PBD duration

**Figure 1:**
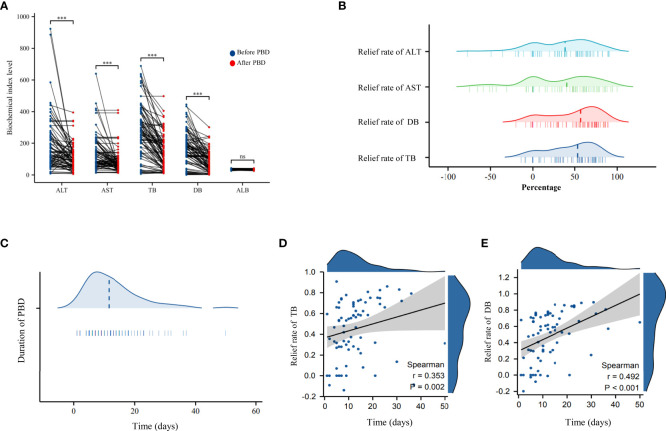
**(A)** Scatter plot of the changes in biochemical indicators before and after biliary drainage in the PBD group. **(B)** Ridge plots showing the reduction in transaminase and bilirubin levels in the PBD group after the use of biliary drainage to reduce jaundice. **(C)** Ridge plots showing the duration of biliary drainage in the PBD group. **(D)** Scatter plot and trendline of the overall rate of reduction in the TB level as a function of the duration of PBD. **(E)** Scatter plot and the trendline showing the overall rate of reduction in DB level as a function of the duration of PBD. PBD, preoperative biliary drainage; TB, total bilirubin (TB); DB, direct bilirubin. *** P < 0.001; ns, no significance.

The time interval from PBD to PD was defined as the duration of biliary drainage, which ranged from 1 day to 50 days (median, 1.5 days; mean, 12.85 days; standard deviation, ± 8.87 days). The efficiency in jaundice reduction or improvement in liver function was calculated as follows: biochemical indicator level before PBD – the most recent biochemical indicator level before PD/biochemical indicator level before PBD.In the PBD group, a significant improvement was noted in TB, DB, AST, and ALT levels (improve efficiency between 38.66%–56.95%) after biliary drainage, which was indicative of a partial relief of the cholestasis and restoration of liver function. The efficiency of jaundice reduction improved with increasing duration of PBD (p < 0.05, **Figures 1A–E**).

### Impact of PBD duration and jaundice reduction efficiency on post-PD complications

**Figure 2:**
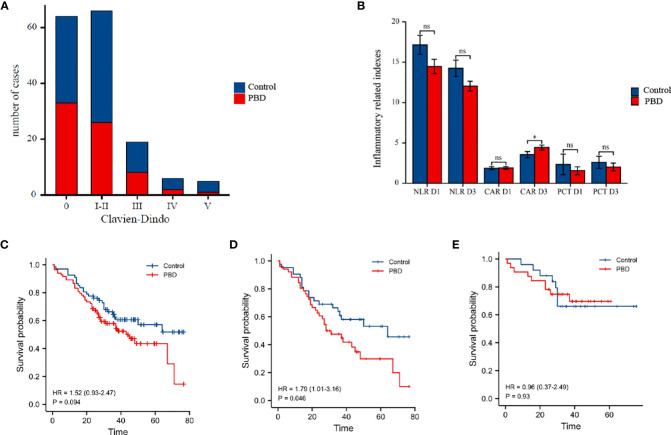
**(A)** Box plot of the number of postoperative complications in the PBD versus control group, according to the Clavien-Dindo classification of the severity of complication. **(B)** Histogram comparing postoperative inflammatory indicators in the PBD and control group. **(C)** Comparison of the overall survival curves for the PBD and control group over a 5-year follow-up period in all patients. **(D)** Comparison of the overall survival curves for the PBD and control group over a 5-year follow-up period in patients with pancreatobiliary type carcinomas. **(E)** Comparison of the overall survival curves for the PBD and control group over a 5-year follow-up period in patients with intestinal type adenocarcinoma.PBD,preoperative biliary drainage. *P < 0.05; ns for not statistically.

The overall rate of complications was 60% (96/160): 65.55% (59/90) in the PBD group and 52.86% (37/70) in the control group. The overall rate of severe complications was 18.75% (30/160): 21.11% (19/90) in the PBD group and 15.71% (11/70) in the control group, with no significant between-group difference noted. The overall rate of grade B/C pancreatic fistula as a complication was 20% (32/160): 21.11% (19/90) in the PBD group and 18.57% (13/70) in the control group. The overall rate of intra-abdominal or gastrointestinal bleeding was 20% (32/160): 23.33% (21/90) in the PBD group and 15.71% (11/70) in the control group. The overall rate of gastric emptying disorder was 16.25% (26/160): 17.77% (16/90) in the PBD group and 14.28% (10/70) in the control group. The overall rate of intra-abdominal infection was 15% (24/160): 14.44% (13/90) in the PBD group and 15.71% (11/70) in the control group (**Figure 2A**). No significant between-group difference was noted for each complication reported (all p>0.05, **Table 2**). In order to eliminate confounding factors and more accurately assess the impact of biliary drainage itself on postoperative complications, we performed logistics regression analysis on the risk factors of postoperative pancreatic fistula.The results suggested that the diameter of pancreatic duct was a risk factor for postoperative pancreatic fistula(OR=0.597,p=0.013,**Table 3**). Then we included these risk factors as covariates to conduct logistics regression analysis on the occurrence of postoperative complications. The results suggested that the histology of tumor was a risk factor for postoperative complications (OR=2.17, p=0.048), while preoperative biliary drainage (OR=1.77, p=0.709, **Table 4**) and other included factors were not risk factors for postoperative complications. In binary logistics regression analysis of severe postoperative complications, the results suggested that preoperative biliary drainage was still not a risk factor (OR=1.89, p=0.388, **Table 5**). In order to analyze the effect of preoperative biliary drainage on postoperative complications, patients in the PBD group were divided into anterograde group and retrograde group, and compared with the control group, the results showed that there was no significant difference in the incidence of complications (p > 0.05, **Tables S1**, **S2**).

**Table 2:** Complications between PBD group and control group.

	Control group (n = 70)	PBD group (n = 90)	Statistic	P value
Post-operative complication				
No	33	31	3.5277	0.473
Clavien-Dindo I-II	26	40		
Clavien-Dindo III	8	11		
Clavien-Dindo IV	2	4		
Clavien-Dindo V	1	4		
Pancreatic fistula				
No	32	44	2.5109	0.473
A	25	27		
B	10	10		
C	3	9		
Intra-abdominal/gastrointestinal bleeding				
No	59	69	1.428	0.232
Yes	11	21		
Gastric emptying disorder				
No	60	74	0.3528	0.552
Yes	10	16		
Intra-abdominal infection				
No	59	77	0.0498	0.823
Yes	11	13		

**Table 3:** Logistic regression analysis of risk factors of postoperative pancreatic fistula in all patients.

	β	Wals	P value	OR	95% C.I.	
					lower limit	upper limit
Age	-0.02	0.433	0.511	0.98	0.923	1.041
BMI	0.209	3.654	0.056	1.232	0.995	1.526
Histology	1.051	2.114	0.146	2.861	0.694	11.796
Differentiation	-0.07	0.01	0.921	0.932	0.232	3.741
PBD	-0.816	1.585	0.208	0.442	0.124	1.575
CA199	0	0.005	0.943	1	0.998	1.002
Pancreas texture	-0.6	1.303	0.254	0.549	0.196	1.537
Diameter of pancreatic duct	-0.516	6.161	0.013	0.597	0.397	0.897
Operation time	-0.003	0.641	0.423	0.997	0.989	1.005

**Table 4:** Logistic regression analysis of risk factors of complications in all patients.

	β	Wals	P value	OR	95% C.I.	
					lower limit	upper limit
Age	-0.015	0.357	0.55	0.976	0.926	1.029
BMI	0.003	0.002	0.964	0.968	0.822	1.139
Histology	1.028	3.92	0.048	2.171	0.725	6.505
Differentiation	-0.307	0.367	0.545	0.747	0.237	2.354
PBD	0.186	0.139	0.709	1.77	0.62	5.052
CA199	0.453	0.867	0.352	1	0.998	1.002
Diameter of pancreatic duct	-0.209	3.325	0.068	0.841	0.667	1.06
Preoperative level of TB	0	0	0.996	1	0.993	1.007
Operation time	0.007	3.169	0.075	1.007	0.998	1.015

**Table 5:** Logistic regression analysis of risk factors of serious complications in all patients.

	β	Wals	P value	OR	95% C.I.	
					lower limit	upper limit
Age	-0.019	0.273	0.601	0.982	0.916	1.052
BMI	0.016	0.018	0.894	1.016	0.806	1.281
Histology	0.473	0.39	0.533	1.605	0.363	7.084
Differentiation	-0.16	0.039	0.844	0.852	0.173	4.205
PBD	0.638	0.747	0.388	1.892	0.445	8.038
CA199	0.002	2.8	0.094	1.002	1	1.003
Diameter of pancreatic duct	-0.067	0.228	0.633	0.935	0.711	1.23
Preoperative level of TB	0.002	0.158	0.691	1.002	0.993	1.01
Operation time	0.004	0.826	0.364	1.004	0.996	1.011

We further analyzed the effect of the duration of PBD and biliary drainage efficiency on postoperative complications. For a drainage duration of <1 week, there were no significant differences in the overall rate of complications nor the overall rate of severe complications, pancreatic fistula, and grade B/C pancreatic fistula, compared with that noted in the control group (p>0.05, **Table 6**). In contrast, for a drainage duration >2 weeks, the overall rate of complications was higher for the PBD subgroup than for the control group (87.09% *vs* 56.14%, p=0.013, **Table 6**), as was the rate of severe complications (33.33% *vs* 14.03%, p=0.03, **Table 6**). The efficiency of jaundice reduction in terms of lowering the TB and DB levels did not correlate with either the overall rate of complications or the overall rate of pancreatic fistula (both p>0.05, **Table S3**). However, the CAR on postoperative day 3 was significantly higher in the PBD group than in the control group (4.45 *vs* 3.56, p=0.043), with no significant between-group difference noted in other inflammatory indicators (all p>0.05, **Table S4** and **Figure 2B**).

**Table 6:** Relationship between duration time of PBD and postoperative complications.

	PBD≤7 days	PBD>7 days	statistic	p value	PBD<14 days	PBD≥14 days	statistic	p value
Post-operative complication								
No	21	10	0.024	0.87	25	6	6.102	**0.013**
Yes	39	20			32	27		
Serious complications								
No	46	25	0.533	0.46	49	22	4.673	**0.03**
Yes	14	5			8	11		
Postoperative pancreatic fistula(POFP)								
No	28	16	0.355	0.55	29	15	0.245	0.619
Yes	32	14			28	18		
Postoperative pancreatic fistula(POFP)								
No	55	26	0.556	0.45	51	30	0.047	0.826
Yes	5	4			6	3		

Bold values is for the P < 0.05.

### Survival analysis and predictive factors

The mean survival time was 52.24 ± 3.54 months for the control group and 43.61 ± 2.45 months for the PBD group (**Table S5**), with no significant between-group difference (HR=0.67, p=0.104, **Figure 2C**). According to the different histology, the patients were divided into two groups(pancreatobiliary type and intestinal adenocarcinoma type), K-M curves were drawn respectively. The results of subgroup analysis showed that in patients with pancreatobiliary type, the survival time of patients in PBD group was shorter(HR=1.79, p=0.046, **Figure 2D**). However, the survival time of patients with intestinal adenocarcinoma type in PBD group did not change significantly (HR=0.96, p=0.93, **Figure 2E**). On univariate Cox regression analysis, tumor differentiation, histology, CA199, serious complications and pancreatic fistula were retained as independent risk factors of patient survival (all p<0.05). On multivariate Cox regression analysis, tumor differentiation and preoperative CA199 level were still retained as independent risk factors of patient survival (all p<0.05). PBD was not a risk factor for patient survival in both regression analysis (p=0.094, **Table 7**).

**Table 7:** Univariate-multivariate analysis of survival after PD.

Characteristics	Total(N)	Univariate analysis		Multivariate analysis	
		Hazard ratio (95% CI)	P value	Hazard ratio (95% CI)	P value
Gender	150				
Male	88	Reference			
Female	62	1.100 (0.679-1.781)	0.698		
Age	150	1.014 (0.987-1.042)	0.315		
Differentiation	150				
Well/Moderate	108	Reference			
Poor	42	3.323 (2.037-5.420)	**<0.001**	2.441 (1.318-4.521)	**0.005**
Histology	150				
pancreatobiliary type	93	Reference			
intestinal adenocarcinoma type	57	0.489 (0.282-0.846)	**0.011**	0.596 (0.299-1.187)	0.141
Preoperative biliary drainage	150				
No	67	Reference			
Yes	83	1.517 (0.931-2.470)	0.094	1.574 (0.906-2.736)	0.107
CA199	132	1.001 (1.001-1.002)	**<0.001**	1.001 (1.000-1.001)	**0.03**
Serious complications	150				
No	125	Reference			
Yes	25	1.821 (1.027-3.230)	**0.04**	1.902 (0.917-3.945)	0.084
Pancreatic fistula	150				
No	71	Reference			
Yes	79	0.611 (0.378-0.986)	**0.044**	0.598 (0.339-1.057)	0.077

The meaning of the bold values is the p values of the statistical results of these factors were all less than 0.05, with significant statistical difference.

## Discussion

Ampullary malignancies often lead to obstructive jaundice. Accumulation of DB, concomitant with other associated conditions of jaundice, such as biliary tract infection, abnormal liver function, and decreased nutritional status, increases the risk of perioperative complications and poor tumor prognosis (8, 9).

PD, considered as the primary surgical modality for the treatment of ampullary masses, plays a key role in the treatment of resectable ampullary adenocarcinoma (10). Although PD has the disadvantages of wide resection, large trauma, and high difficulty for organ reconstruction, perioperative mortality and the rate of complications associated with PD have decreased in recent years. A clinical study of 28,888 patients, who were recruited across 700 hospitals participating in the American College of Surgeons National Surgical Quality Improvement Program from 2006 to 2019, reported a decrease in the rate of PD-related rate of major complications and mortality within the 30-day perioperative period from 2.5% in 2006 to 1.6% in 2019, although the overall rate of complications increased annually, reaching a rate of 22.76% in 2019 (11). Therefore, controlling perioperative complications, in combination with early diagnosis and treatment of post-PD complications, is critical and should be addressed. In this regard, gaining insights into the risk factors of post-PD complications remains an important clinical task in the long run.

PBD for the treatment of obstructive jaundice was introduced by Whipple in 1935 (12) and was performed as a two-step procedure comprising gallbladder-stomach anastomosis to relieve cholestasis in the first step, followed by resection of the stage II tumor in the second step. With technical advances in the development of ERCP and PTC (13, 14), more biliary drainage methods have been emerging, with PBD becoming a systemic treatment modality for patients with ampullary tumors (15). Since the publication of the results of the DROP trial in 2010 (16), the rate of PBD in patients with pancreaticobiliary tumors has decreased significantly, with focus placed on the optimal treatment strategy following jaundice reduction and treatment modalities for patients who are not suitable for early surgery. However, the results of the DROP trial do not fully discount the clinical significance of PBD. Moreover, the DROP trial itself has some limitations, which need to be acknowledged, such as including only patients with a TB level of 40–250 μmol and using a 7-Fr plastic stent under ERCP for jaundice reduction; these stents predispose the duct to occlusion, leading to a significant increase in the incidence of ERCP-related cholangitis and other complications. Unfortunately, the trial did not elaborate on surgery-related complications. In view of the above issues, the present study was intended to investigate the impacts of PBD on the incidence of PD-related complications and the prognosis.

Subgroup analysis identified that PBD led to a significant decrease in bilirubin and transaminase levels. Moreover, increasing the duration of biliary drainage increased the efficiency of cholestasis relief. The following mechanisms likely contributed to the improvement in patients’ perioperative condition with PBD. First, restoration of the bile level in the intestinal lumen allows bile salts to bind endotoxin, improving intestinal integrity and restoring the composition of the intestinal flora, thereby reducing the incidence of inflammation (17). Second, biliary drainage causes a cessation in the accumulation of hydrophobic bile acid in the liver, significantly reducing hepatocellular injury and improving hepatocellular function (18). Third, patients with malignant tumors combined with obstructive jaundice are in a hypercoagulable state (i.e., having an abnormal coagulation function); biliary drainage can improve the coagulation factor levels and the fibrinolytic process and can even correct this pathological state (19). Through these mechanisms, PBD may provide a strong pathophysiological benefit in patients with ampullary tumors and cholestasis.

The current first-line method for PBD is ERCP, which can produce some inflammatory irritation to the ampullary sphincter, lower common bile duct, pancreatic head, and surrounding areas (20, 21). Inflammation can increase exudation and adhesions in these anatomical structures, which potentially increases the surgical risk (22, 23). Thus, although PBD can relieve cholestasis and improve liver function to repair hepatocytes, PBD may increase the risk of complications during PD surgery. Therefore, the impact of PBD on post-PD complications deserves more attention. In our study, although the rate of postoperative complications was slightly higher in the PBD group than in the control group, this difference was not significant. This finding likely reflects the decline in inflammatory status at the surgical site, ultimately after biliary drainage. Moreover, relief of cholestasis combine with nutritional support after PBD and the associated adjustment of the body’s internal environment increase the efficiency of post-PD recovery (24).

The time interval from PBD to PD remains a key issue to address. A previous study has shown that when undergoing biliary drainage, 3–6 weeks are needed for the body to restore hepatocellular signaling, β-oxidation, and mitochondrial function (25). Therefore, it has been widely accepted that biliary drainage should be performed for 4–6 weeks prior to PD (26, 27). At our center, the average duration of PBD in patients with malignant biliary obstruction is 12 days. In this study, we compared outcomes based on a duration of PBD of <1 week or >2 weeks. While no significant difference was noted in the overall rate of post-PD complications between the PBD group who underwent a drainage period of <1 week and the control group, the rate of post-PD complications and severe complications was significantly higher after a duration of biliary drainage of >2 weeks. It is possible that patients requiring prolonged biliary drainage may have more comorbidities, a worse nutritional status, and a worse physiological baseline and that a prolonged duration of biliary drainage may increase the systemic inflammatory response. The relative changes in the TB and DB levels after biliary drainage did not correlate with the rate of complications, probably because bilirubin indicators alone neither reflect the degree of cholestasis relief nor the degree of hepatocyte recovery.

This study has some limitations. The relatively small sample size of our study must also be acknowledged in this regard. More indicators of liver function and cholestasis, such as AST, ALT, ALB, GGT, and ALP, may be screened in future studies to identify a comprehensive indicator that can reflect the efficiency of biliary drainage.

Whether PBD affects the survival of patients is also an issue of high concern. While the excessive delay in surgical treatment can result in tumor progression, affecting survival, there is currently no consensus as to whether incomplete drainage may be beneficial, with absence of accepted guidelines for the optimal duration of PBD (28, 29). A high-quality multicenter randomized controlled trial reported a mean survival of 12.2 months after biliary endoscopic sphincterotomy compared with the 12.7 months with PBD; this difference not being significant (30). The findings of our study were consistent with those of this trial, with a mean survival of 52 months for the control group and 43 months for the PBD group (**Table 7**); this difference not being significant. Although PBD did not provide survival benefits, it is not an inferior treatment option as short-term drainage can improve PD surgery and recovery by promoting the functional reserve and homeostasis of the liver without adversely affecting survival.

## Conclusion

PBD neither affects the incidence of post-PD complications nor the survival in patients who have developed both ampullary malignancies and biliary obstruction. Prolonged biliary drainage (>2 weeks) may increase the overall rate of postoperative complications and severe complications. General surgery clinicians should perform PBD in a more selective, targeted, and individualized manner for such patients. Moreover, PD surgery should be performed only when both cholestasis and hepatocellular function can be improved, as to avoid an increased risk of surgery-related complications due to the prolonged duration of biliary drainage. The most appropriate comprehensive treatment strategy should be selected based on individual patient characteristics.

## Data availability statement

The raw data supporting the conclusions of this article will be made available by the authors, without undue reservation.

## Ethics statement

The studies involving human participants were reviewed and approved by ethics committee of Beijing Friendship Hospital, Capital Medical University (approval number: 2022-P2-104-01). This study complies with the Declaration of Helsinki, and all patients obtained informed consent before being included in this clinical study. Written informed consent for participation was not required for this study in accordance with the national legislation and the institutional requirements.

## Author contributions

WG, HL and DW contributed to conception and design of the study. PL, CG and XZ organized the database. XY ZF and CX performed the statistical analysis. HL and DW wrote the first draft of the manuscript. YM and XG wrote sections of the manuscript. All authors contributed to the article and approved the submitted version.

## Conflict of interest

The authors declare that the research was conducted in the absence of any commercial or financial relationships that could be construed as a potential conflict of interest.

## Publisher’s note

All claims expressed in this article are solely those of the authors and do not necessarily represent those of their affiliated organizations, or those of the publisher, the editors and the reviewers. Any product that may be evaluated in this article, or claim that may be made by its manufacturer, is not guaranteed or endorsed by the publisher.
